# Evolutionary Dynamics and Complicated Genetic Transmission Network Patterns of HIV-1 CRF01_AE among MSM in Shanghai, China

**DOI:** 10.1038/srep34729

**Published:** 2016-10-04

**Authors:** Xiaoshan Li, Yile Xue, Yi Lin, Jing Gai, Lei Zhang, Hua Cheng, Zhen Ning, Leiming Zhou, Kexin Zhu, Guido Vanham, Laiyi Kang, Ying Wang, Minghua Zhuang, Qichao Pan, Ping Zhong

**Affiliations:** 1 Department of AIDS and STD, Shanghai Municipal Center for Disease Control and Prevention; Shanghai Municipal Institutes for Preventive Medicine, Shanghai, China; 2 School of Public Health, Southeast University, Nanjing, China; 3 Research Center for Public Health, School of Medicine, Tsinghua University, Beijing, China; 4 School of Public Health, Nantong University, Nantong, China; 5 Biomedical Sciences Department, Institute of Tropical Medicine of Antwerp, Belgium

## Abstract

To explore the evolutionary dynamics and molecular transmission patterns of HIV-1 CRF01_AE in depth among men who have sex with men (MSM) in Shanghai, we constructed phylogenetic tree and genetic transmission networks based on 1, 152 *pol* sequences from MSM, 282 from other risk groups and 795 references. Phylogenetic analyses identified two distinct major CRF01_AE lineages and a Shanghai-based sub-lineage. The estimated tMRCAs for lineage 1 and 2 were 1996.0 (1992.9–1999.2) and 1997.8 (1994.3–2001.4), respectively. Of the 1, 152 MSM, 681 (59.1%) were identified as belonging to 241 separate networks. Of these 681 individuals in networks, 74.2% were linked to cases diagnosed in different years, 4.3% were linked to heterosexual women, and 0.7% were linked to persons who inject drugs. A total of 71 networks including 180 individuals diagnosed in Shanghai with the same domicile were found. Recent infection (*P* = 0.022) and sampling year after 2011 (*P* < 0.001) were significantly associated with potential transmission links among the networks. Besides, a significant transmission of viruses with drug resistant mutations at V179D/E were found in the networks. Given these findings, we propose that genetic transmission analysis is a useful tool in HIV intervention strategies to curb the spread of virus and promoting public health.

HIV-1 CRF01_AE has become the most prevalent strains in China^1^, especially in men who have sex with men (MSM)^1^,^2^,^3^. In our studies, based in Shanghai, we have previously confirmed a high percentage of CRF01_AE strains among MSM^4^,^5^. Moreover, we showed a low-level baseline CD4 + T cells, a high CXCR4 co-receptor usage and a rapid loss of CD4 + T cells leading to progression to AIDS among CRF01_AE-infected individuals, as compared to those who were infected with CRF07_BC^4^,^5^,^6^. However, the viral genetic evolution, population dynamics and patterns of transmission of CRF01_AE among MSM remained largely unknown in Shanghai.

Comprehensive surveillance strategies are necessary to monitor the rapid spread of HIV among MSM. However, the silence and secrecy associated with societal stigma and discrimination as well as the clinical latency of HIV-1 are barriers for timely surveillance of HIV-1 transmission among this population. The recent advancement in genetic transmission network analyses techniques, together with the quickly expanding HIV sequence database represent an opportunity to better understand temporal and spatial transmission characteristics^7^. In addition, the combined analysis of genetic, demographic and epidemiological data may provide a useful approach for elucidating the transmission pattern of HIV^8^,^9^.

In the present study, we describe the molecular evolutionary dynamics of HIV-1 CRF01_AE among Shanghai MSM using a phylogenetic inference analysis based on 1, 152 *pol* gene sequences from Shanghai HIV-1-infected MSM and 282 other risk groups’ sequences derived from 2008 through 2013, and 795 CRF01_AE reference strains. We then re-constructed the relatively deeply sampled sub-networks of CRF01_AE to depict the temporal and spatial transmission of networks and explore the linking-associated factors among genetic transmission networks.

## Results

### Socio-demographic characteristics of Shanghai CRF01_AE-infected MSM

A total of 1, 152 MSM infected with HIV-1 CRF01_AE, newly diagnosed in Shanghai between 2008 and 2013, were enrolled in this retrospective study. The group had an average age of 31.45 ± 9.63 years, with a predominance of young MSM i.e. 832/1, 152 or 72.2% were <35 years. All participants were Han ethnicity, and 64.5% (743/1, 152) were domestic migrants. Approximately half (50.3%, 579/1, 152) had a college or higher (≥13 years) level of education. Most were single (71.5%, 824/1, 152). Almost one third (32.3%, 372/1, 152) had ≥6 sex partners in the past 6 months. Median CD4 + T cell counts in the first follow-up (<3 months after infection confirmation) was 360.9 (IQR: 238.3–499.0) cells/μl. These demographic data are consistent with other reports in Shanghai^10^,^11^,^12^,^13^.

### Multiple introductions of CRF01_AE strains into Shanghai MSM

**Figure 1:**
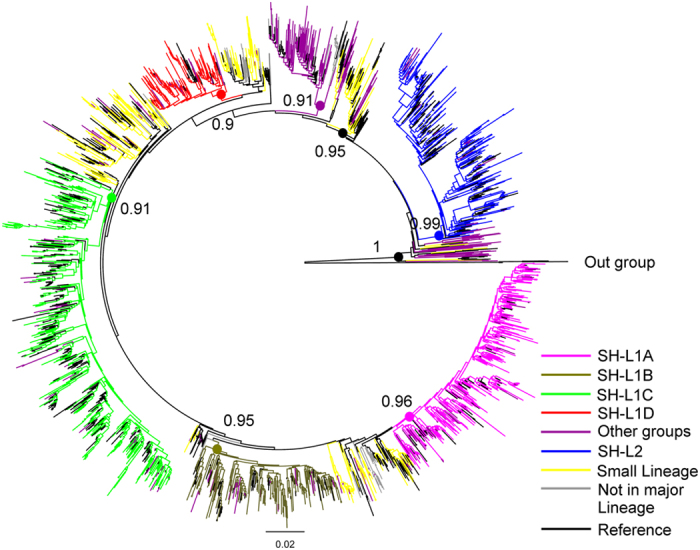
Phylogenetic analysis of lineages/sub-lineages. The phylogenetic tree was constructed using approximately-maximum-likelihood method based on *pol* region (HXB2: 2, 253 to 3, 306 nt) in FastTree 2.3. The nucleotide substitution mode was GTR + G + I. The bootstrap value ≥0.9 was identified as a lineage/sub-lineage and was indicated at all relevant nodes. HIV-1 subtype C was chosen as an out-group in the rooted tree. The various lineages/sub-lineages were color-coded.

Bayesian skyline plot (BSP) deduced that CRF01_AE strains among Shanghai MSM started its initial growth phase around 1998, followed by an exponential expansion during 2000–2007, and reached a steady growth afterwards (Supplementary material 1). Phylogenetic analysis clearly identified two distinct major lineages, Shanghai lineage 1 (SH-L1) and Shanghai lineage 2 (SH-L2) (constituent ratio: 87.2% and 12.4%, Fig. 1). The estimated time of the most recent common ancestors (tMRCAs) for SH-L1 and SH-L2 were 1996.0 (1992.9–1999.2) and 1997.8 (1994.3–2001.4), respectively (Supplementary material 2). The estimated mean evolutionary rate was 2.7 × 10^−3^ nucleotide substitutions/site/year. The demographic characteristics did not differ substantially between two lineages (Supplementary material 3). SH-L1 consisted of four independent sub-lineages (SH-L1A-D), while SH-L2 just presented a small monophyletic lineage (Fig. 1). A dozen other minor lineages were dispersed in the phylogenetic tree. Previously seven distinct CRF01_AE phylogenetic lineages were found nationwide by China CDC^14^. Remarkably, three of the SH-L1 sub-lineages (except 1D) clustered together with China-lineage 4, while SH L2 clustered together with China-lineage 5. However, we did not find any of the other Chinese lineages in our cohort^14^ (Supplementary material 4). Of interest was that the sub-lineage 1D, accounting for 8.6% (n = 99), did not match with any reported lineages in China, indicating a unique Shanghai-based epidemic. The estimated tMRCA for this new identified sub-lineage was 2003.5 (2001.0–2005.9), later than all other sub-lineages (Supplementary material 2). Compared to other lineages/sub-lineages, the individuals included in 1D were more likely to be single (82.8% versus 68.5%, *P* = 0.028), while other demographic characteristics did not differ.

### Identification and characterization of genetic transmission networks

**Figure 2:**
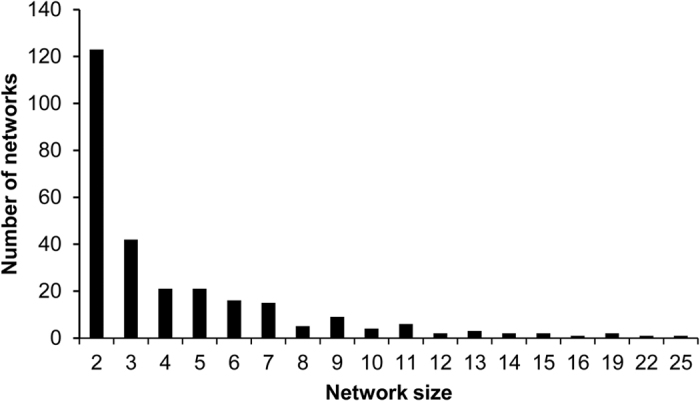
Distribution of HIV-1 CRF01_AE genetic transmission networks among MSM in Shanghai.

**Figure 3:**
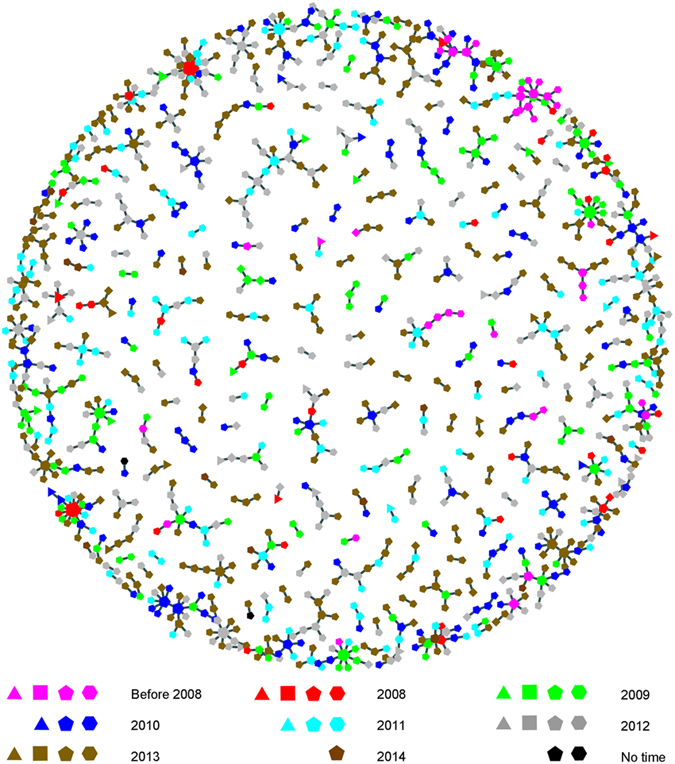
Sampling year-associated and group-associated genetic transmission networks. Different colors represent different years. Different shapes represent different groups: triangle: heterosexual women; square: heterosexual men; pentagon: MSM; hexagon: unknown.

Of 2, 229 individuals, 1, 211 (54.3%) were segregated into 276 networks (Supplementary material 5), with sizes ranging between 2 and 25, (Fig. 2), in which 241 networks were related to 681 Shanghai MSM individuals (Fig. 3). Of all networks, 123 (44.6%) were made up of only 2 individuals. The number of networks was inversely correlated to network size (Spearman’s correlation coefficient = −1.0, *P* < 0.001), Fig. 2.

Of the 681 Shanghai MSM in networks, 74.2% (505) were linked to cases diagnosed in different years. As shown in Fig. 3, individuals newly diagnosed in different years were clearly interlinked. Of the 241 networks related to Shanghai MSM, 53 (including 88 Shanghai MSM individuals) were composed of cases diagnosed in same years, while 188 (including 593 Shanghai MSM individuals) were composed of cases diagnosed in different years. The proportions of linkage between individuals diagnosed in different years were 78.8% (2008), 60.2% (2009), 86.6% (2010), 72.4% (2011), 66.9% (2012) and 57.8% (2013), respectively, indicating a high transmission over the years. Of the 681 Shanghai MSM in networks, 88.1% (600/681) were linked to other MSM, 4.3% (29/681) were linked to heterosexual women, 0.7% (5/681) were linked to persons who inject drugs (PWID), and 10.9% (74/681) were linked to heterosexual men.

To explore the potential transmission between recent infection and long-standing infection in the networks, 517 (out of 681, 75.9%) recent infections (<1 year) were identified using a molecular algorithm (See Methods), of which 417 (80.7%) were interlinked among recent infections, and 162 (31.3%) were linked to the long-standing infections (>1 year). Of the 164 long-standing infection, 71 (43.3%) were interlinked among long-standing infections, and 117 (71.3%) were linked to recent infections.

**Figure 4:**
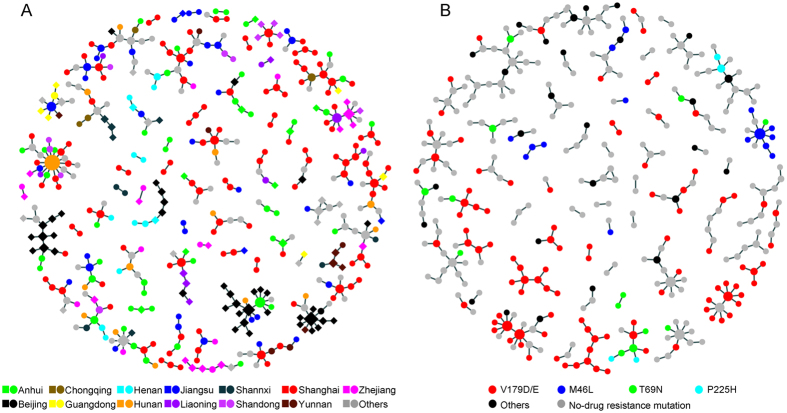
Domicile places-associated genetic transmission networks (**A**) and Drug- resistant-associated genetic transmission networks (**B**). In Fig. 4A, square represents individuals diagnosed in other provinces/cites, and dot represents individuals diagnosed in Shanghai. Different colors represent different domicile places. In Fig. 4B, different colors represent different drug mutations.

To better understand the role that the migrants (living in Shanghai for more than half a year) played in the formation of networks, we investigated the influence of their domicile on transmission. We found the transmission linkage existed not only in the individuals with different domiciles and diagnosed in Shanghai (intra-province), but also in the individuals with same domiciles (townsmen) but diagnosed in the domicile places and Shanghai (inter-province). Seventy-one networks including 180 individuals diagnosed in Shanghai (dots in different colors based on domicile) and those who were diagnosed in their hometowns (squares in different colors based on domicile) are shown in Fig. 4A and Supplementary material 6. In addition, 5 networks including 11 individuals diagnosed in Shanghai were linked with 6 Thai individuals (Supplementary material 7).

Overall, 40.0% (461/1, 152) of persons had 1 link, and 19.1% (220/1, 152) of persons had ≥2 links in our network analysis. Chi-square test (Table 1) showed that, sampling year after 2011 (*P* < 0.001) and recent infection (*P* = 0.022) were factors associated with potential transmission links among networks. These persons with ≥2 links were involved in 474 (69.6%) persons through multiple links in the established genetic transmission network. Of note, 17 individuals with ≥5 links were involved in 112 (16.4%) persons (Fig. 2).

### Drug resistant-associated genetic transmission networks

Among the 1, 152 studied individuals, 59 (5.1%) harbored drug resistance mutations conferring resistance to at least one class of antiretroviral (ARV) drugs. Only one individual harbored three mutations including M46I, A62V and T69N. The rate of drug resistant mutation did not differ between sampling years (from 2008 to 2013, 8.0%, 7.4%, 3.7%, 6.8%, 5.2%, 4.2%, respectively; *P* = 0.441). Overall, four main network-related drug resistant mutations (some were non-tansmitted drug resistance mutations) were discovered at V179D/E (n = 53), M46L (n = 15), T69N (n = 8), and P225H (n = 2), Fig. 4B. Clearly, Among 1, 152 Shanghai CRF01_AE, the proportion of V179D/E was 7.1% (82/1, 152), higher than all other China CRF01_AE (2.7%, 90/3, 291), *P* < 0.001. V179D/E mutation distributed in 26 networks in lineage 1B (n = 8), 1C (n = 33), lineage 2 (n = 3), and small lineage (n = 9). Only 2 networks for M46L, 6 networks for T69N, and 1 network for P225H were found in lineage 1C and 1B, lineage 1D, 1B, 1 A, small lineage, lineage1D, and lineage 1D, respectively.

## Discussion

In this study, we identified two major CRF01_AE lineages circulating among Shanghai MSM individuals and a new sub-lineage that was unreported elsewhere in China. The SH-L1 included most of subjects’ sequences (87.2%) and had a close match with China-lineage 4, the most common CRF01_AE lineage in MSM epidemic nationwide, while SH-L2 was closely associated with China-lineage 5, a common lineage related to heterosexuals and injection drug users epidemic^14^. Our previous study and others indicated that about 20% MSM in Shanghai had sex with women and an estimated 8.3% MSM in China was reported to have used illicit drugs in the past 6 months. In this study, the genetic transmission network analysis reconfirmed the interaction between MSM and other groups. Although the introduction of SH-L1 into Shanghai MSM was slightly earlier than SH-L2, it showed an obviously evolutionary divergence of CRF01_AE which had resulted in at least 4 sub-lineages epidemics. We speculate that the new sub-lineage would probably continue to expand given 60.6% of its members are domestic migrants.

As a coastal international metropolis, Shanghai attracted an increasing numbers of domestic migrant people in recent years. As these migrants constantly face difficulties in accessing employment, social welfare, education, and health services locally under the current household registration system, they usually flow between hometowns and different cities^15^. Our network analysis revealed the occurrence of transmission not only inside Shanghai city, but also between Shanghai and other provinces, indicating the complicated transmission patterns and the difficulty in intervention among MSM population. We realize that ongoing HIV infection and the high proportion of networking may also reflect a concentrated uncontrolled MSM epidemic in Shanghai, due to lack of diagnosis, non-treatment in time after diagnosis, failure of viral suppression after ART initiated, and lack of contact with care^11^,^16^,^17^,^18^. In this study, we found that recent infection was closely related to potential transmission linking among networks, which is consistent with previous studies, suggesting that early HIV-1 infection has an important role in transmission events indeed^19^. This observation, once again, emphasizes the importance of early diagnosis and timely antiretroviral treatment. Previous studies showed that the individuals with more links in the network have a higher probability of spreading the virus to others because of a high viral load and a high rate of partner change^20^,^21^, and therefore, these individuals may play the role of super-spreaders^22^,^23^. Thus, our, genetic transmission network analysis confirmed that a minority of super-spreaders indeed drive transmission networks to a significant extend.

An interesting observation in this study was that V179D/E, an NNRTI-associated mutation was dominating Shanghai MSM. Sequences with V179D/E were distributed and networked in most sub-lineages in SH-L1, suggesting that several independent CRF01_AE strains with V179D/E were involved in ongoing transmission in this city. Besides, we found a higher proportion of V179D/E mutation in Shanghai CRF01_AE strains than in all others China CRF01_AE strains. This observation constitutes an alternative molecular evidence for determination of HIV-1 transmission among Shanghai MSM.

Although the present study was intended to include all first-time follow-up MSM after diagnosed, there was still ~20% loss to follow. Besides a misclassification as heterosexuals could be present among MSM^24^. However, analysis of ≥50% subjects from the available follow-up subjects in this study was suggested to satisfy the requirements of cluster analysis in the concentrated areas^25^. Therefore, we believed that the size of samples derived from MSM was reasonable and might not significantly bias our analysis.

In conclusion, we elucidated the molecular evolutionary dynamics of HIV-1 CRF01_AE circulating among Shanghai MSM. Genetic transmission network analysis further revealed a complexity of transmission pattern. We thus suggest that network analysis based on a molecular approach, combined with social science and public health approaches could be helpful in effective HIV intervention strategy to curb the spread of virus and promote public health.

## Methods

### Study subjects

A total of 2, 252 MSM blood samples were collected at the first follow-up after HIV diagnosis during 01/2008-12/2013, representing >80% of newly HIV-1 diagnosed MSM in Shanghai. A total of 1, 836 sample sequences were acquired after RNA extraction and PCR amplification, among which, 1, 152 CRF01_AE isolate sequences with >1, 000 *bp* were selected for further analysis. The corresponding demographic characteristics and CD4 + T cell counts were also collected for analysis. None of the individuals was exposed to antiretroviral treatment (ART) at the time of enrolment. In addition, 130 heterosexual women sequences, 134 heterosexual men sequences and 18 PWID sequences were acquired from individuals diagnosed in Shanghai. We also isolated all closely related publicly available sequences with 1, 152 CRF01_AE strains of MSM from HIV databases of Los Alamos National Laboratory. We performed a BLAST search^26^ by using the 1, 152 sequences, and selected top 10 sequences with the highest homology with the references for each sequence. A total of 795 reference sequences were selected for analysis after excluding repeated sequences. Eventually, 2, 229 CRF01_AE sequences were included for subsequent analysis. In order to exclude the influence of convergent evolution at drug resistance mutation site on the phylogenetic analysis, 45 sites in protease (PR) and reverse transcriptase (RT) were removed before phylogenetic analysis. HIV-1 Drug resistance mutations were determined using Stanford University HIV Drug Resistance Database tool: HIVdb Program: Sequence Analysis (http://sierra2.stanford.edu/sierra/servlet/JSierra?action=sequenceInput) and the last updated (Oct-Nov 2015) guidelines from the International AIDS Society Resistance Testing-USA panel^27^. HIV-1 transmitted drug mutations (TDR) were determined based on the WHO 2009 list of mutations for surveillance of TDR HIV strains (http://hivdb.stanford.edu/pages/WHOResistanceList.html).

### Phylogenetic analyses

FastTree 2.3^28^ was used to estimate an approximately-maximum likelihood phylogenetic tree for *pol* sequences using the GTR + G + I nucleotide substitution model. The phylogenetic tree’s reliability was determined with local support values based on the Shimodaira-Hasegawa (SH) test^29^ and presented using FigTree v1.3.1 (http://beast.bio.ed.ac.uk). Monophyletic groups with bootstrap support ≥0.9 were identified as a lineage.

### Bayesian skyline plot analysis and divergence time estimation

Bayesian skyline plot (BSP) analysis was conducted to explore the changes in the effective population size of CRF01_AE among MSM over time in Shanghai. The models selected were GTR + Relaxed clock (uncorrelated) + Bayesian skyline. The tMRCAs of CRF01_AE lineages were estimated using a Bayesian inference approach to explore the timescale of CRF01_AE expansion among Shanghai MSM. Rates of evolution (in units of nucleotide substitutions/site/year) were estimated simultaneously. Phylogenies were inferred using BEAST v.1.7.2. The Markov chain Monte Carlo (MCMC) analysis was computed for 200 million generations and sampled every 1,000 steps and output was assessed for convergence by means of effective sampling size (ESS) after a 20% burn-in using Tracer. To minimize the effects of standard errors, only ESS ≥200 were accepted.

### Identification and analysis of genetic transmission networks

The flowchart of genetic transmission networks was depicted as Supplementary material 8. Briefly, transmission clusters were extracted from the phylogenetic tree using the software Cluster Picker^30^. Transmission clusters were defined as node support threshold greater than 95% and intra-cluster maximum pairwise genetic distances less than 3.0% nt substitutions per site. As a significant proportion of the sequences (26.3%) were presumably from patients in the stage of long-standing infection (details in below), we chosen a threshold of genetic distance confined to 3.0%^31^,^32^,^33^, in order to identify relevant transmission clusters^30^. The pairwise genetic distances of all sequences within the available clusters were calculated. The minimum genetic distances algorithm was used to define the linkages within a cluster (Supplementary material 9). For visualizing and analyzing network, the network data were processed using a custom R script utilizing the network package in the R software^34^.

### Analysis of individuals with potential transmission Links

Three groups were compared including (1) individuals with no link to others, (2) individuals who linked to another one, and (3) individuals who linked to ≥2 others. Chi-square test was used to determine linking-associated factors among networks. The demographic and laboratory information retrieved included sampling year, recent infection, age, domicile, education, marital status, number of sex partner in the past 6 months, and CD4 + T cell counts. We used a molecular algorithm of a frequency of ambiguous calls in bulk sequencing of *pol* gene under 0.5% to define a recent infection event <1 year prior to clustering analysis^35^,^36^.

### Ethics statement

The study protocol was reviewed and approved by the Institutional Review Board at the Human Medical Research Ethics Committee of the Shanghai CDC. No additional informed consent from participants was obtained for this special investigation as the data were analyzed retrospectively and anonymously. All research methods in this study were carried out in accordance with the approved guidelines.

## Additional Information

**How to cite this article**: Li, X. *et al*. Evolutionary Dynamics and Complicated Genetic Transmission Network Patterns of HIV-1 CRF01_AE among MSM in Shanghai, China. *Sci. Rep.* **6**, 34729; doi: 10.1038/srep34729 (2016).

## Supplementary Material

Supplementary Information

## Figures and Tables

**Table 1:** Factors associated with Potential Transmission Links.

**Characteristics**	**Total**	**0 Link, n (%)**	**1 Link, n (%)**	**≥2 Links, n (%)**	**χ**^**2**^	***P***
Sampling Year					25.394	<0.001
2008–2011	512	243(47.5)	164(32.0)	105(20.5)		
2012–2013	640	228(35.6)	297(46.4)	115(18.0)		
Recent infection (<1 year)					7.678	0.022
Yes	844	327(38.7)	357(42.3)	160(19.0)		
No	308	144(46.8)	104(33.8)	60(19.5)		
Domicile					2.879	0.578
Locals	388	159(41.0)	151(38.9)	78(20.1)		
Migrants	743	300(40.4)	304(40.9)	139(18.7)		
Unknown	21	12(57.1)	6(28.6)	3(14.3)		
Age					0.077	0.962
<35 years	832	342(41.1)	331(39.8)	159(19.1)		
≥35 years	320	129(40.3)	130(40.6)	61(19.1)		
Education					3.646	0.456
1–12 years	546	223(40.8)	224(41.0)	99(18.1)		
≥13 years	579	233(40.2)	228(39.4)	118(20.4)		
Unknown	27	15(55.6)	9(33.3)	3(11.1)		
Marital status					11.786	0.067
Singlehood	824	341(41.4)	319(38.7)	164(19.9)		
Married	153	62(40.5)	67(43.8)	24(15.7)		
Divorced or widowed	130	42(32.3)	62(47.7)	26(20.0)		
Unknown	45	26(57.8)	13(28.9)	6(13.3)		
CD4 + T cell counts (cells/μl)					1.226	0.542
<350	557	231(41.5)	227(40.8)	99(17.8)		
≥350	595	240(40.3)	234(39.3)	121(20.3)		
Number of sex partner in the past 6 months					2.355	0.671
<5	630	265(42.1)	244(38.7)	121(19.2)		
≥6	372	152(40.9)	153(41.1)	67(18.0)		
Unknown	150	54(36.0)	64(42.7)	32(21.3)		
