# Supplementary material for: Evolutionary Dynamics and Complicated Genetic Transmission Network Patterns of HIV-1 CRF01_AE among MSM in Shanghai, China
Source: Sci Rep. 2016 Oct 4;6:34729. doi: 10.1038/srep34729 (PMC5048130; doi:10.1038/srep34729)
Supplement: Supplementary Information [file srep34729-s1.doc]

**Evolutionary Dynamics and Complicated Genetic transmission network Patterns of HIV-1 CRF01_AE among MSM in Shanghai, China**

Xiaoshan Li^1,2#^, Yile Xue^1#^, Yi Lin^1#^, Jing Gai^1#^, Lei Zhang^3#^, Hua Cheng^1#^, Zhen Ning^1^, Leiming Zhou^1^, Kexin Zhu^4^, Guido Vanham^5^, Laiyi Kang^1^, Ying Wang^1^, Minghua Zhuang^1^, Qichao Pan^1*^ and Ping Zhong^1*^

^1^ Department of AIDS and STD, Shanghai Municipal Center for Disease Control and Prevention; Shanghai Municipal Institutes for Preventive Medicine, Shanghai, China; ^2^ School of Public Health, Southeast University, Nanjing, China; ^3^ Research Center for Public Health, School of Medicine, Tsinghua University, Beijing, China; ^4^ School of Public Health, Nantong University, Nantong, China; ^5^ Biomedical Sciences Department，Institute of Tropical Medicine of Antwerp, Belgium

# These authors contributed equally to this work.

*Corresponding author.

E-mail: zhongp56@hotmail.com (P.Z.); panqichao@scdc.sh.cn (Q.P.)

**Supplementary material 1.** **Bayesian skyline plot of CRF01_AE strains among Shanghai MSM.** Molecular clock analysis was performed using BEAST v1.7. Baysian skyline plot output was analyzed using Tracer v1.5. Markov Chain Monte Carlo (MCMC) chains was run at least 200 million generations and sampled every 1000 steps. The x-axis represents time in years, and the y-axis represents the effective population size. The thick solid line is the mean estimates and the 95% HPD credible region is shown by blue areas.


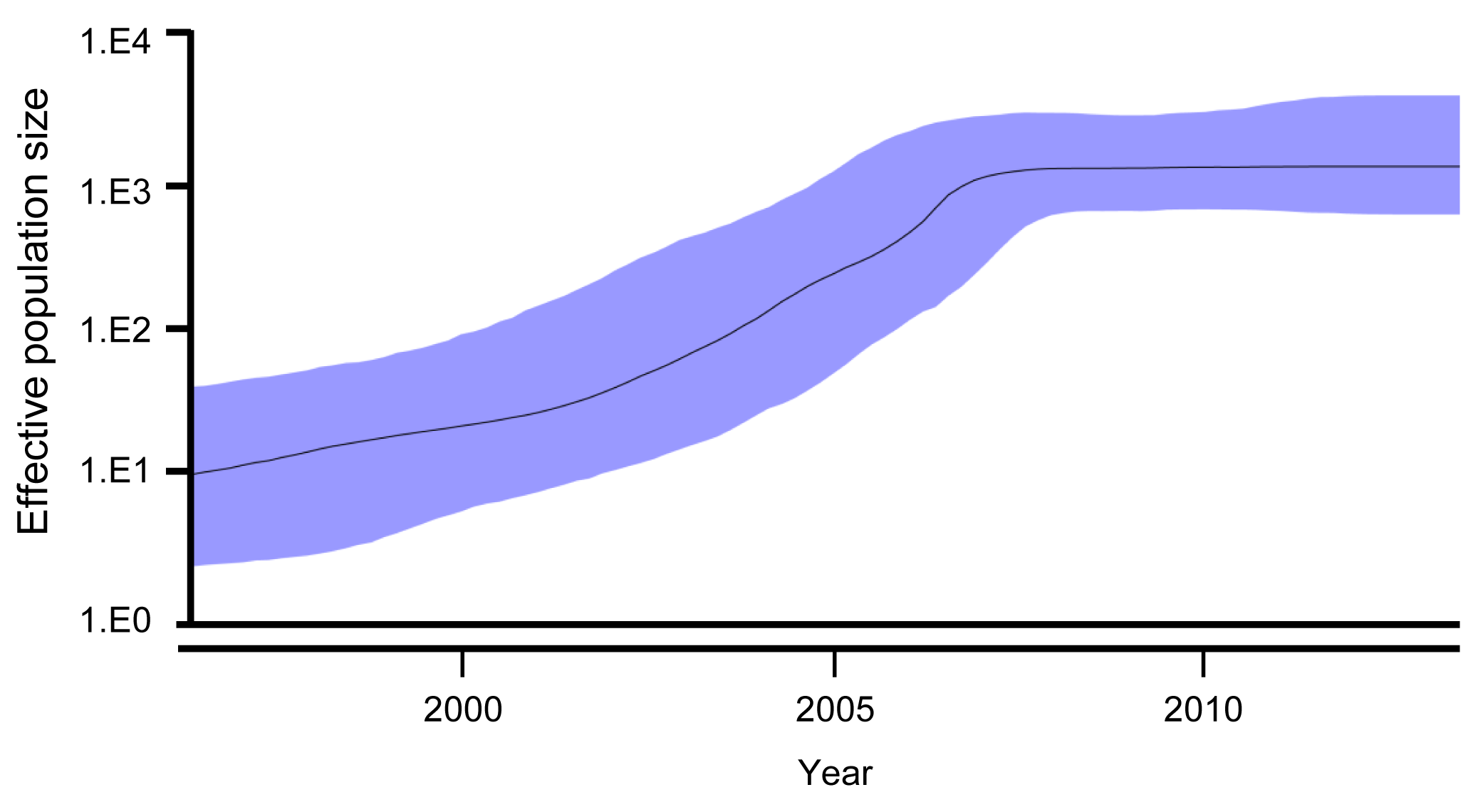


**Supplementary material 2. Estimated time of the most recent common ancestors (tMRCAs) of CRF01_AE for** **each lineage.**

|  | GTR+γ4+BSP | |  | HKY+γ4+BSP | |  | GTR+γ4+constant size | |  | HKY+γ4+constant size | |
| --- | --- | --- | --- | --- | --- | --- | --- | --- | --- | --- | --- |
|  | Relaxed | Strict |  | Relaxed | Strict |  | Relaxed | Strict |  | Relaxed | Strict |
|  | µ^a^=2.65  (2.17-3.3) | µ=2.55  (2.11-2.95) |  | µ=2.64  (2.14-3.09) | µ=2.53  (2.12-2.96) |  | µ=2.26  (1.85-2.69) | µ=2.19  (1.79-2.54) |  | µ=2.22  (1.83-2.67) | µ=2.15  (1.77-2.53) |
|  | ESS^b^=1124 | ESS=1349 |  | ESS=937 | ESS=1590 |  | ESS=1080 | ESS=1097 |  | ESS=1240 | ESS=1032 |
|  | tMCA^c^ | tMCA |  | tMCA | tMCA |  | tMCA | tMCA |  | tMCA | tMCA |
| African | 1974.4  (1967.3-1981.5) | 1974.8  (1970.1-1979.4) |  | 1974.4  (1967.5-1981.3) | 1974.5  (1970.1-1979.0) |  | 1972.9  (1966.0-1980.0) | 1972.9  (1967.8-1977.9) |  | 1972.8  (1965.7-1979.8) | 1973.1  (1967.9-1978.2) |
| Thailand | 1984.4  (1980.2-1988.5) | 1984.8  (1981.7-1987.8) |  | 1984.4  (1980.5-1988.3) | 1984.6  (1981.5-1987.7) |  | 1983.3  (1979.0-1987.6) | 1983.4  (1976.2-1990.6) |  | 1983.1  (1978.8-1987.4) | 1983.5  (1980.0-1987.1) |
| SH-L1 | 1996.0  (1992.9-1999.2) | 1996.2  (1993.6-1998.7) |  | 1996.1  (1992.9-1999.3) | 1996.0  (1993.1-1998.8) |  | 1995.4  (1992.1-1998.6) | 1995.3  (1992.4-1998.1) |  | 1995.1  (1991.6-1998.6) | 1995.3  (1992.4-1998.3) |
| SH-L1A | 2001.1  (1998.7-2003.5) | 2001.1  (1999.1-2003.1) |  | 2001.0  (1998.6-2003.5) | 2000.8  (1998.8-2002.7) |  | 2000.2  (1997.7-2002.6) | 2000.0  (1997.7-2002.3) |  | 1999.9  (1997.2-2002.6) | 2000.0  (1997.8-2002.2) |
| SH-L1B | 1999.6  (1996.7-2002.4) | 1999.5  (1997.1-2001.9) |  | 1999.6  (1996.7-2002.5) | 1999.2  (1996.9-2001.6) |  | 1999.8  (1996.9-2002.7) | 1999.7  (1997.1-2002.3) |  | 1999.6  (1996.6-2002.6) | 1999.8  (1997.1-2002.5) |
| SH-L1C | 2002.0  (1999.8-2004.2) | 2001.8  (2000.0-2003.7) |  | 2002.0  (1999.9-2004.1) | 2001.6  (1999.7-2003.5) |  | 2001.2  (1998.6-2003.7) | 2000.8  (1998.4-2003.1) |  | 2000.9  (1998.3-2003.5) | 2000.8  (1998.5-2003.2) |
| SH-L1D | 2003.5  (2001.0-2005.9) | 2003.0  (2000.9-2005.1) |  | 2003.5  (2001.1-2005.9) | 2002.8  (2000.7-2004.9) |  | 2003.22  (2000.9-2005.6) | 2002.8  (2000.6-2005.0) |  | 2003.0  (2000.7-2005.3) | 2002.8  (2000.6-2005.1) |
| SH-L2 | 1997.8  (1994.3-2001.4) | 1998.0  (1995.4-2000.6) |  | 1997.8  (1994.3-2001.2) | 1997.7  (1995.1-2000.3) |  | 1996.1  (1992.5-1999.8) | 1995.8  (1992.6-1999.1) |  | 1995.8  (1992.0-1999.6) | 1995.9  (1992.7-1999.0) |

^a^ Estimates of the mean evolutionary rate (μ×10^–3^ nucleotide substitutions/site/year) for the CRF01_AE.

^b^ ESS: Effective sampling size.

^c^ Mean time of the most recent common ancestor (tMRCA: year) for the CRF01_AE dataset (95% HPD in parentheses).

**Supplementary material 3. Socio-demographic characteristics of studied participants based on two major lineages.**

| **Characteristic** | **Total** | **Lineage 1,**  **n (%)** | **Lineage 2,**  **n (%)** | **χ^2^** | ***P*** |
| --- | --- | --- | --- | --- | --- |
| **Sampling Year** ^a^ |  |  |  | 0.005 | 0.942 |
| 2008-2011 | 509 (44.3) | 446 (44.4) | 63 (44.1) |  |  |
| 2012-2013 | 639 (55.7) | 559 (55.6) | 80 (55.9) |  |  |
| **Recent infection (<1 year)** ^a^ |  |  |  | 1.070 | 0.301 |
| Yes | 842 (73.3) | 732 (72.8) | 110 (76.9) |  |  |
| No | 306 (26.7) | 273 (27.2) | 33 (23.1) |  |  |
| **Domicile** ^b^ |  |  |  | 0.960 | 0.619 |
| Locals | 387 (33.7) | 341 (33.9) | 46 (32.2) |  |  |
| Migrants | 740 (64.5) | 647 (64.4) | 93 (65.0) |  |  |
| Unknown | 21 (1.8) | 17 (1.7) | 4 (2.8) |  |  |
| **Age (years)** ^a^ |  |  |  | 0.182 | 0.670 |
| <35 | 828 (72.1) | 727 (72.3) | 101 (70.6) |  |  |
| ≥35 | 320 (27.9) | 278 (27.7) | 42 (29.4) |  |  |
| **Education (years)** ^b^ |  |  |  | 2.516 | 0.284 |
| 1-12 | 546 (47.6) | 481 (47.9) | 65 (45.5) |  |  |
| ≥13 | 575 (50.1) | 503 (50.0) | 72 (50.3) |  |  |
| Unknown | 27 (2.4) | 21 (2.1) | 6 (4.2) |  |  |
| **Marital status** ^a^ |  |  |  | 2.988 | 0.393 |
| Singlehood | 821 (71.5) | 726 (72.2) | 95 (66.4) |  |  |
| Married | 153 (13.3) | 133 (13.2) | 20 (14.0) |  |  |
| Divorced or widowed | 130 (11.3) | 110 (10.9) | 20 (14.0) |  |  |
| Unknown | 44 (3.8) | 36 (3.6) | 8 (5.6) |  |  |
| **CD4+ T cell counts (cells/µL)** ^a^ |  |  |  | 0.339 | 0.560 |
| <350 | 556 (48.4) | 490 (48.8) | 66 (46.2) |  |  |
| ≥350 | 592 (51.6) | 515 (51.2) | 77 (53.8) |  |  |
| **Number of sex partner**  **in the past 6 months** ^a^ |  |  |  | 1.532 | 0.465 |
| <5 | 630 (54.9) | 545 (54.2) | 85 (59.4) |  |  |
| ≥6 | 372 (32.4) | 329 (32.7) | 43 (30.1) |  |  |
| Unknown | 146 (12.7) | 131 (13.0) | 15 (10.5) |  |  |

^a^ Chi-square test; ^b^ Fisher’s exact test

**Supplementary material 4. The comparison of Shanghai CRF01_AElineages and China CRF01_AE lineages firstly reported by China CDC.** The phylogenetic tree was constructed using approximately-maximum-likelihood method based on *pol* region (HXB2: 2253 to 3306 nt) in FastTree 2.3. The nucleotide substitution mode was GTR+G+I. The various lineages/sub-lineages were color-coded. The China-lineages were firstly reported by China CDC. HIV-1 subtype C was chosen as an out-group in the rooted tree.


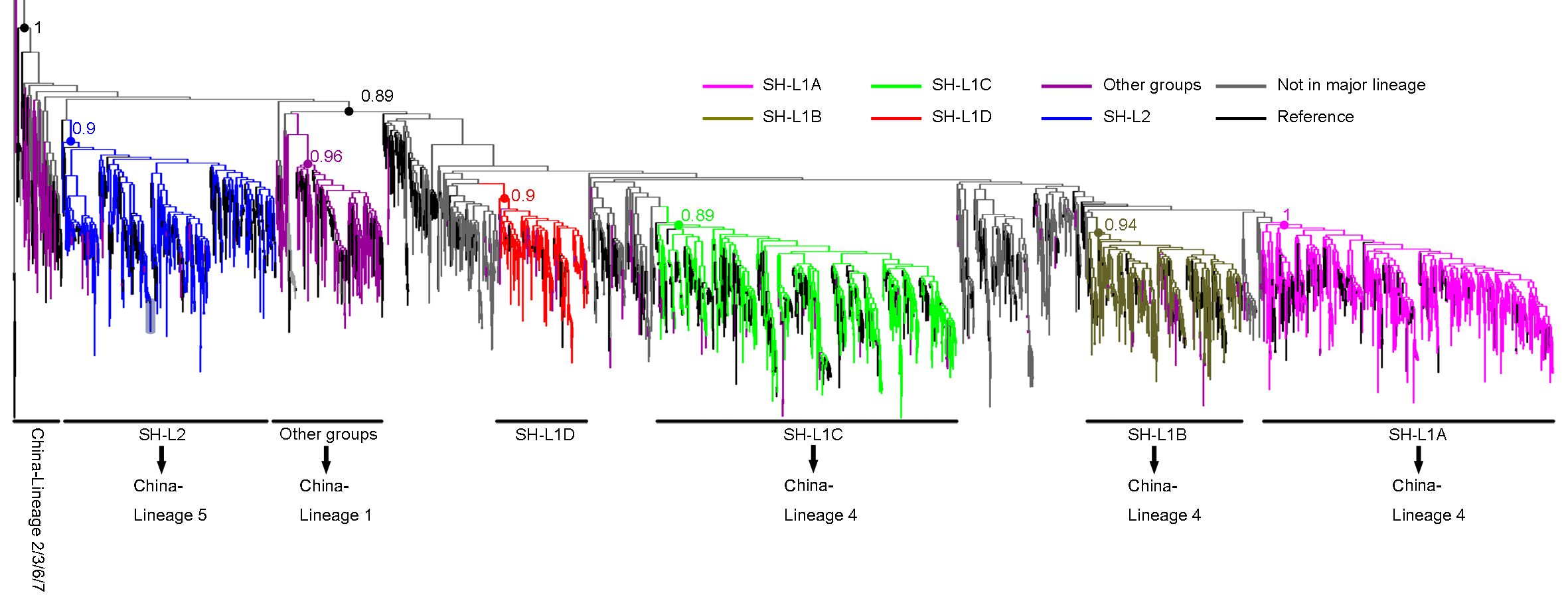


**Supplementary material 5. Depiction of various terms used to describe the network.** (Alexandra M. Oster, et al. Using Molecular HIV Surveillance Data to Understand Transmission Between Subpopulations in the United States. J Acquir Immune Defic Syndr, 2015, 70(4) : 444-451.)

**
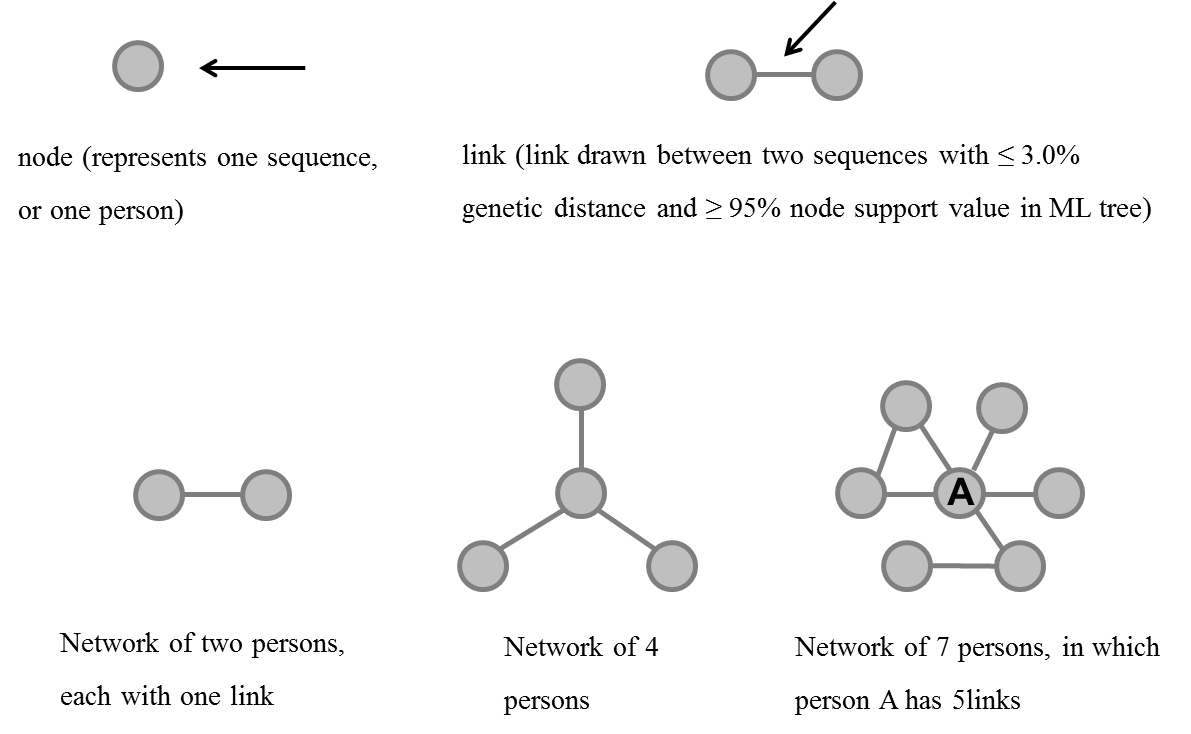
**

**Supplementary material 6.** **The genetic transmission networks including Shanghai individuals with the same domiciles.** SH: Diagnosed in Shanghai and domiciles is Shanghai; AH (SH): Diagnosed in Shanghai and domiciles is Anhui; AH: Diagnosed in Anhui; JS (SH): Diagnosed in Shanghai and domiciles is Jiangsu; JS: Diagnosed in Jiangsu; ZJ (SH): Diagnosed in Shanghai and domiciles is Zhejiang; ZJ: Diagnosed in Zhejiang; HEN (SH): Diagnosed in Shanghai and domiciles is Henan; HEN: Diagnosed in Henan; GD (SH): Diagnosed in Shanghai and domiciles is Guangdong; GD: Diagnosed in Guangdong; SD (SH): Diagnosed in Shanghai and domiciles is Shandong; BJ (SH): Diagnosed in Shanghai and domiciles is Beijing; BJ: Diagnosed in Beijing; CQ (SH): Diagnosed in Shanghai and domiciles is Chongqing; HUN (SH): Diagnosed in Shanghai and domiciles is Hunan; HUN: Diagnosed in Hunan; LN (SH): Diagnosed in Shanghai and domiciles is Liaoning; LN: Diagnosed in Liaoning; SHX (SH): Diagnosed in Shanghai and domiciles is Shannxi; YN (SH): Diagnosed in Shanghai and domiciles is Yunnan; YN: Diagnosed in Yunnan.

**Supplementary material 7. Seven genetic transmission networks were involved in international transmission.**

**
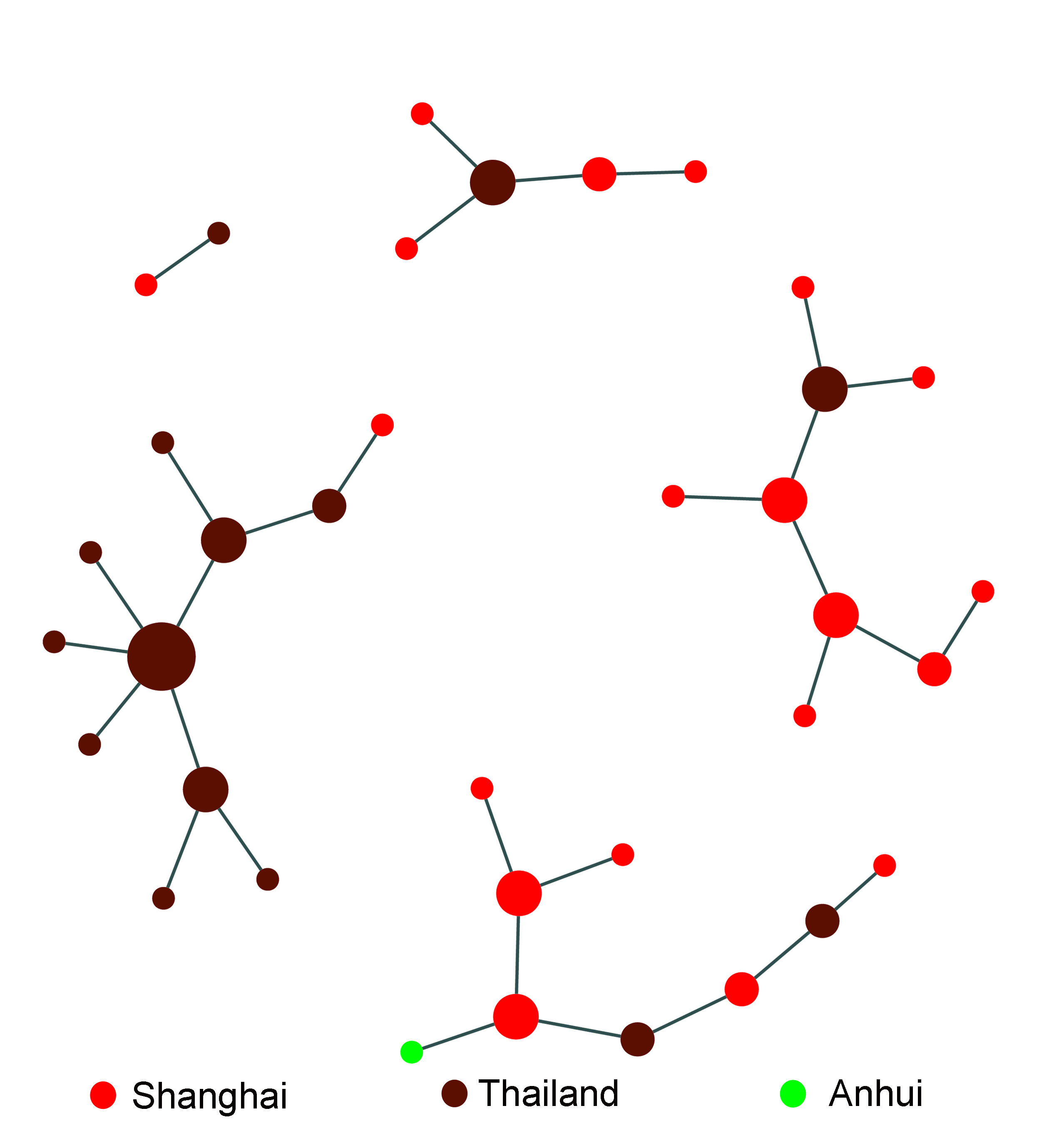
**

**Supplementary****material 8. Flowchart of genetic transmission networks creation.**

**2222 *pol* sequences (1152 Shanghai MSM, 282 Shanghai other groups and 795 references**

**Using FASTTREE construct ML (maximum likehood) tree**

**1211 sequences segregated into 276 clusters**

**Using network package in the R software create networks**

**Remove patients with no links: using CP (CLUSTER PICKER) extract transmission clusters and exclude patients with no links. Molecular transmission clusters were defined as node support threshold greater than 95% and intra-cluster pairwise genetic distances less than 3.0%.**

**The pairwise genetic distances of all sequences within the available clusters were calculated. The minimum genetic distances algorithm was used to decide the linkage among individuals each other within a cluster.**

**Supplementary material 9. The minimum genetic distances algorithm.**

(1) Phylogenetic tree

**
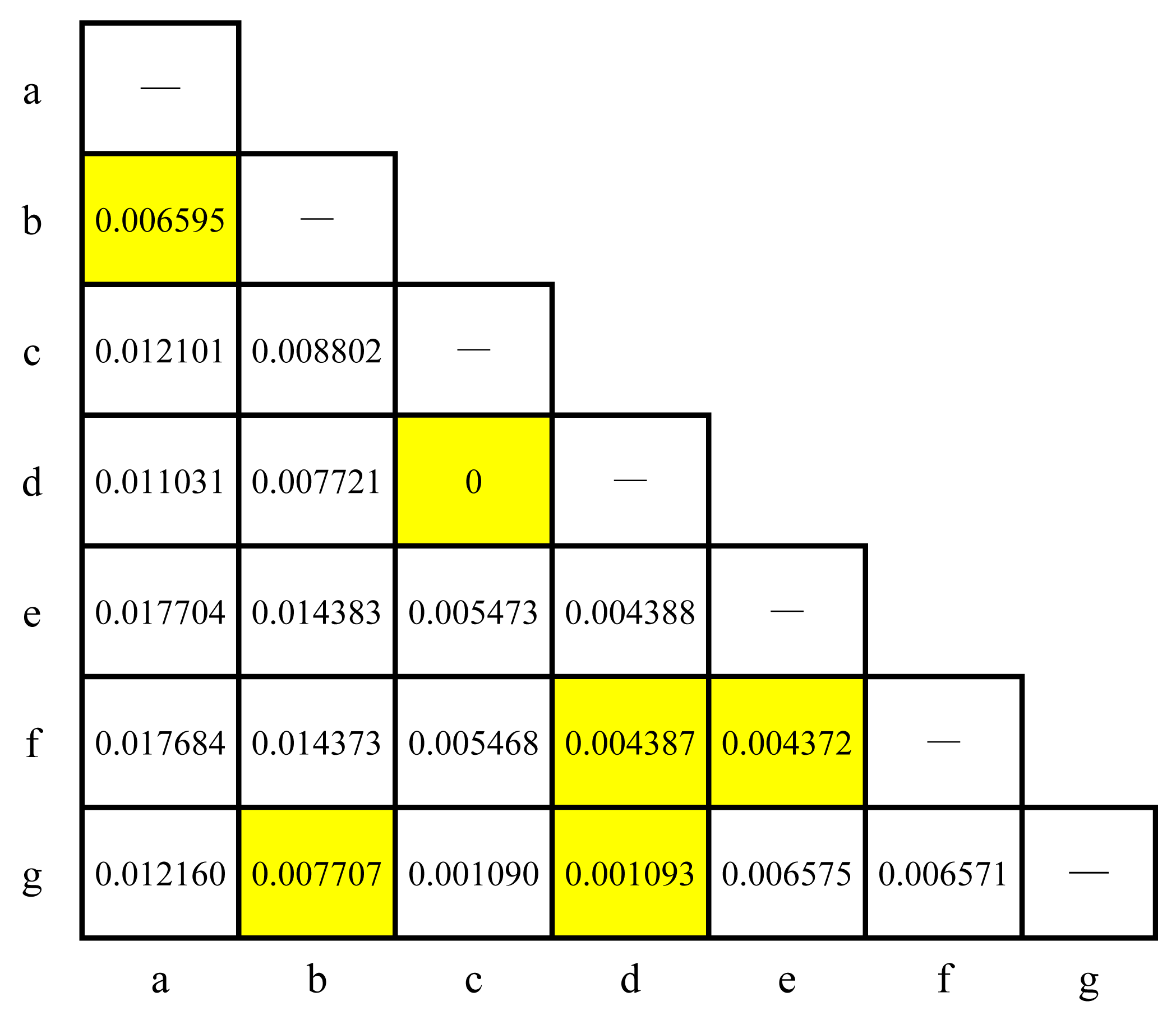
**

(2) Among all undirected trees select one that minimizes the sum of edge weights (genetic distances)

**
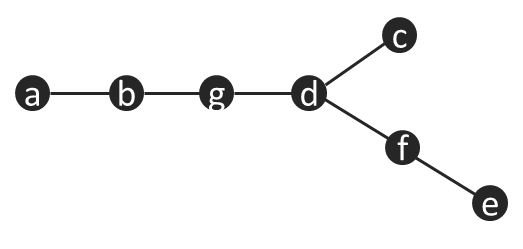
**

(3) Network
